# One-hole split endoscopy technique versus unilateral biportal endoscopy technique for L5-S1 lumbar disk herniation: analysis of clinical and radiologic outcomes

**DOI:** 10.1186/s13018-023-04159-9

**Published:** 2023-09-09

**Authors:** Yuhong Zhang, Bo Feng, Peng Hu, Guohua Dai, Weiliang Su

**Affiliations:** 1 Department of Spine Surgery, Binzhou Medical University Hospital, No. 661, Huanghe Er Road, Binzhou, 256603 Shandong China; 2 Department of Neurology, Binzhou Medical University Hospital, No. 661, Huanghe Er Road, Binzhou, 256603 Shandong China

**Keywords:** One-hole split endoscopy technique, Unilateral biportal endoscopy technique, Lumbar disk herniation, Efficacy

## Abstract

**Background:**

Lumbar disk herniation (LDH) is one of the most common diseases of the spine, especially occurring in L4-5 and L5-S1 intervertebral disks, and surgery is a choice when conservative treatment is ineffective. The purpose of this study is to investigate the clinical efficacy and radiologic outcomes of one-hole split endoscopy (OSE) technique versus unilateral biportal endoscopy (UBE) technique in the treatment of L5-S1 lumbar disk herniation (LDH).

**Methods:**

A total of 133 patients of a single center surgically treated for L5-S1 LDH between 2019 and 2021 were retrospectively included in this study, of which 70 were treated by UBE technique and the rest were treated by OSE technique. Hospitalization time, operative time, intraoperative blood loss, fluoroscopy times, incision length and related complications were recorded. Bone resection area (BRA), articular process resection rate, range of motion (ROM), sagittal translation (ST), disk height (DH), Visual Analog Score (VAS), Oswestry Disability Index (ODI) and Macnab criteria were used to evaluated the clinical efficacy.

**Results:**

There was no statistically significant difference in hospitalization time or fluoroscopy times between the two groups. The operation time was shorter in the UBE group than that in the OSE group; however, the incision length was longer. Intraoperative blood loss and BRA were larger in the UBE group than in the OSE group. There was no significant difference in ROM, ST, DH, or postoperative facet resection rate between the two groups. There was no significant difference in ROM, ST, or postoperative facet resection rate compared with preoperative indicators in each group, but there was a significant difference in DH among distinct groups. At any time point, the lower back and leg VAS and ODI in each group were significantly improved compared to those before the operation, with no significant difference between the two groups. There was one case of dural tear in the UBE group. One case of transient hypoesthesia occurred in each of the two groups. The excellent–good rates of the UBE group and the OSE group were 88.6% and 90.5%, respectively.

**Conclusion:**

The OSE technique is an effective minimally invasive surgical option as well as the UBE technique in the treatment of L5-S1 LDH.

## Introduction

Lumbar disk herniation (LDH) is a common cause of low back and leg pain, and surgery is an effective treatment when conservative treatment is ineffective [1]. Percutaneous endoscopic lumbar discectomy (PELD) has been widely used in the treatment of LDH, and percutaneous endoscopic transforaminal discectomy (PETD) has minimal trauma and rapid recovery and is effective as a commonly used minimally invasive treatment for LDH [2]. In some patients, PETD may be challenging to treat the LDH of L5-S1 segment due to the influence of anatomical factors such as high iliac crest, L5 transverse process hypertrophy, articular process hyperplasia, enlarged lumbosacral angle, high sacral flank and narrow intervertebral foramina [3]. As a minimally invasive technique attracting much attention in recent years, unilateral biportal endoscopy (UBE) technique has a remarkable effect in the treatment of LDH, and a large number of studies have also demonstrated its clinical efficacy [4, 5]. One-hole split endoscopy (OSE) technique is an emerging technique that has been applied clinically in China. Similar to UBE technique, OSE technique has working and observation channels, but the two are located in the same soft incision, operated separately, and each can rotate and swing freely. Our department has treated a subset of patients with lumbar spine diseases using the UBE and OSE techniques. This retrospective study aims to investigate the clinical efficacy and radiologic outcomes of the OSE technique versus UBE technique in the treatment of L5-S1 LDH by comparing with the UBE technique.

## Materials and methods

### Patient characteristics

This was a retrospective study which was in accordance with the ethical standards of the 1964 Declaration of Helsinki and its later amendments. Ethics committee approval and due consent were also obtained.

Among the patients with L5-S1 LDH treated by our department from January 2019 to June 2021, 70 patients treated with UBE technique (the UBE group) and 63 patients treated with OSE technique (the OSE group) who met the inclusion criteria were included in this study. Inclusion criteria were: (i) Imaging results showed L5-S1 LDH which single segment was involved, and clinical symptoms and signs were consistent with imaging findings; (ii) the symptoms of nerve roots of the lower extremities were predominant, with or without low back pain; and (iii) at least 3 months of conservative treatment was ineffective, seriously affecting work and life. Exclusion criteria were: (i) lumbar spine instability or ≥ II° lumbar spondylolisthesis; (i) lumbar kyphosis or scoliosis deformity with Cobb angle ≥ 20°, or congenital anatomical abnormalities; (iii) intervertebral disk diseases such as discitis, intervertebral space infection, tumor, tuberculosis; or (iv) those who had combined psychiatric-related diseases to affect function evaluation. All patients underwent dynamic X-ray, CT and MRI examinations before surgery. All patients were treated by the same surgeon.

### Surgical techniques

#### OSE group

After anesthesia was induced, patients were placed in the prone position on a fluoroscopic surgery table with the abdomen suspended. Sterile cloth was spread after routine disinfection was applied. The endoscopes, RF electrode knife, grinding drill and perfusion system were connected. C‐arm fluoroscopy was used to confirm the target segment. Taking the operation on the left incision as an example, a longitudinal incision approximately 1.2 cm long was made and the portal location was 1.5 cm lateral to the spinous process at the horizontal line of the responsible intervertebral space. Skin, subcutaneous tissue and deep fascia were incised in turn, and the dilator gradually expanded the soft tissue to the bony surface of the lamina for blunt separation. The OSE and operating instruments were inserted into the incision, and the perfusion system was opened. A total of 3000 ml of isotonic saline was selected as the flushing fluid and placed at a level of approximately 50–60 cm above the operation area. After the field of view was cleared by flushing with isotonic saline, a low-temperature plasma radio frequency cutter head was used to handle the interlaminar soft tissue and the surface tissue of the ligamentum flavum, revealing lower margin of the upper vertebrae lamina, upper margin of the lower vertebrae lamina, ligament flavum, root of the spinous process, and medial edge of the superior and inferior articular processes. High-speed dynamic grinding drills and a laminar rongeur were used to remove part of the lamina bone to the attachment point of the ligament flavum and separate the adhesion between the dural sac and the ligament flavum. The left part of the ligament flavum was removed, and the dural sac, nerve roots and intervertebral disk were exposed. Under the endoscope, a nerve retractor was used to protect the dural sac and nerve root from injury, and a nerve dissector was used to peel off the adhesion and explore the free intervertebral disks and annulus fibrosus. A pituitary rongeur was used to remove protruding and loose nucleus pulposus tissue in the intervertebral space. Calcified adhesion around the nerve root was properly excised to ensure full decompression. The left nerve root channel and spinal canal were further enlarged. Finally, radio frequency was applied to shrink the fiber ring fracture and shape. A bipolar RF knife head was used to stop bleeding, the field was rinsed with water, the working sleeve was pulled out, the incision was sutured, and the site was covered with a sterile dressing.

#### UBE group

C-arm fluoroscopy was used to confirm the lower margin of the upper lamina of the responsible intervertebral space. Centered on the intersection of the horizontal line of the lower edge of the lamina and the medial edge of the left upper and lower pedicles as the center, a total of two transverse incisions approximately 12 mm long were made on the superior side and inferior side, and the distance between the two cuts was approximately 30 mm. The working channel and observation channel were placed according to the patient's body size and specific surgical methods. The remainder of the operation was performed in the same manner as in the OSE group.

### Clinical and radiological evaluation

The patient's hospitalization time, operative time, intraoperative blood loss, incision length, fluoroscopy times and related complications were recorded. Intraoperative blood loss was estimated by subtracting the amount of perfusion from total irrigation liquid outflow. Visual Analog Score (VAS) was used to assess the degree of low back pain and leg pain before surgery and at three days, three months, and 18 months after surgery. Score of Oswestry Disability Index (ODI) was used to assess functional improvement before surgery and at three months and 18 months after surgery. Clinical outcomes at 18 months postoperatively were evaluated using the Macnab criteria.

**Fig. 1:**
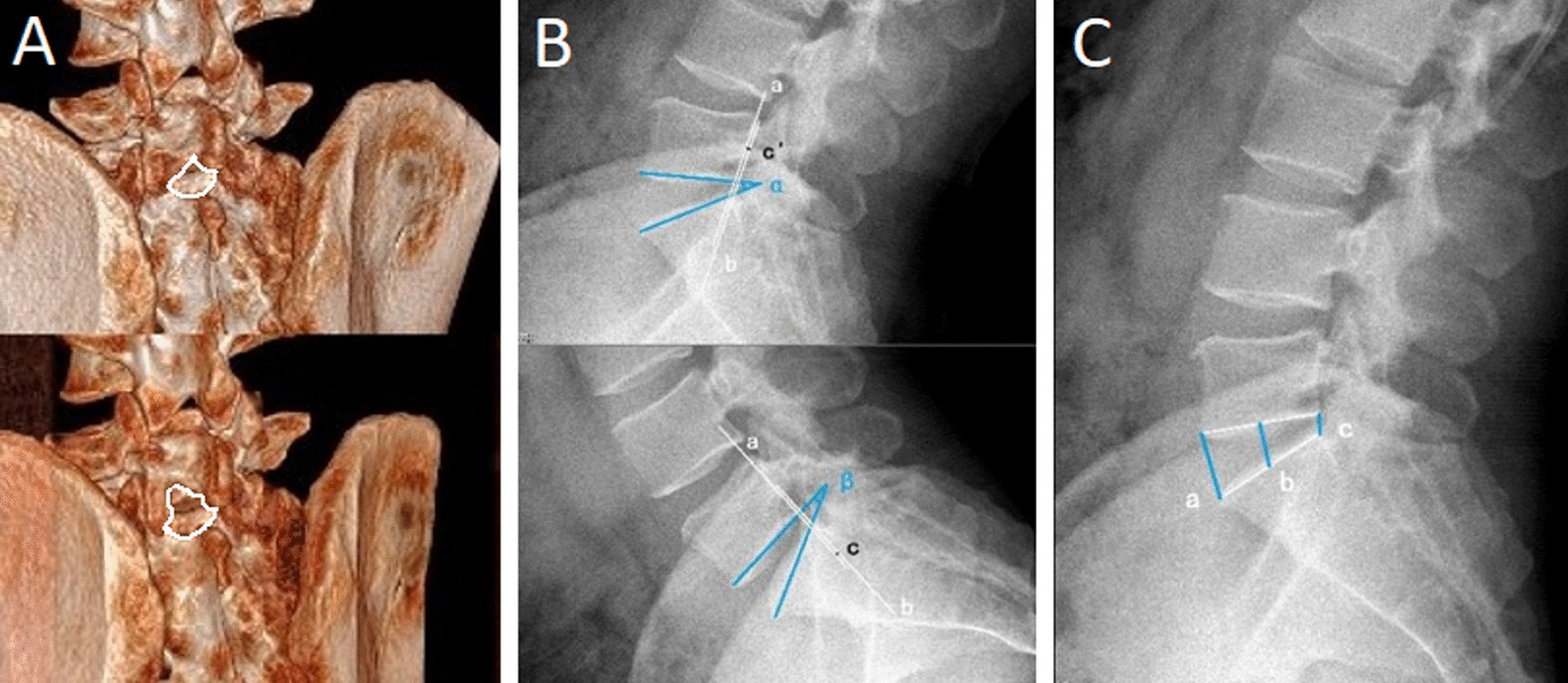
**A** Measure the area of 3D CT lamina space before and after surgery to estimate the area of bone resection, and BRA = postoperative ISA–preoperative ISA. **B** Dynamic position X-ray was used to measure ROM and ST before and after surgery, ROM = α − β, ST =|c–c'|. (**C)** Lateral lumbar X-ray was used to measure DH before and after surgery, DH = (a + b + c)/3

Carestream Vue PACS software (Carestream Health, Canada) was used to measure the interlaminar space area (ISA) at the same location of 3D CT before and after surgery to estimate bone resection area (BRA), and BRA = postoperative ISA–preoperative ISA; the length of the responsible segment in the 3D CT axis articular surface was measured before and after surgery to estimate the articular process resection rate; range of motion (ROM) and sagittal translation (ST) of the surgical segment were measured before surgery and at 18 months after surgery to evaluate the effect of surgery on the stability of the lumbar spine; disk height (DH) was measured before surgery and at 18 months after surgery to assess the impact of surgery on the disks (Fig. 1).

### Statistical analysis

All statistical analyses were performed using SPSS 24.0 software. The χ^2^ test was used for comparison of counting data. The Shapiro‒Wilk test was used to test the normality of the distribution, and values are expressed as the mean (standard deviation) or median (range). The comparison between the two groups was made by independent sample *t* test or nonparametric test. Comparisons of VAS and ODI were analyzed by repeated measurement ANOVA. A *p* value of < 0.05 was accepted as statistically significant.

## Results

Procedures were successfully performed in all of the patients. The demographic statistics for the OSE and UBE groups are reported in Table 1. The two groups did not differ significantly in terms of age, sex, body mass index (BMI), lower extremity symptoms, preoperative VAS of the low back and leg, or ODI. There were 21 cases and 27 cases of giant LDH in the UBE group and the OSE group, respectively, with no statistical significance (*p* > 0.05). There were 31 cases and 22 cases of migrated LDH in the UBE group and the OSE group, respectively, with no statistical significance (*p* > 0.05). There were nine cases and six cases with calcification in the UBE group and the OSE group, respectively, with no statistical significance (*p* > 0.05).

**Table 1:** Baseline characteristics of the two groups

	UBE (*n* = 70)	OSE (*n* = 63)	*p* value
Age, years (SD)	49.1(11.2)	52.1(12.3)	0.145
Female sex-no (%)	28(47)	21(61)	0.107
Body mass index, kg/l^2^ (SD)	24.9(1.2)	24.7(1.4)	0.370
Lower extremity symptoms			0.732
Unilateral lower extremity symptom	54	47	
Bilateral lower extremity symptom	16	16	
Gigantic LDH	21	27	0.123
Migrated LDH	31	22	0.271
High-grade migration type	12	6	0.386
Low-grade migration type	19	16	
Calcified LDH	9	6	0.544
VAS (SD)			
For lower back pain	4.3(1.3)	4.0(1.4)	0.162
For leg pain	7.5(0.7)	7.5(0.6)	0.553
ODI(SD)	63.0(6.3)	60.7(6.9)	0.050

Mean (standard deviation) or number (%) as stated

*VAS* Visual analog scale, *ODI* Oswestry disability index

*p < 0.05, statistical significance

### Clinical outcome

The hospitalization time (*p* = 0.147) fluoroscopy times (*p* = 0.110), and number of excellent-good case (*p* = 0.783) were not significantly different between the two groups. However, the differences in operative time (*p* = 0.001), intraoperative blood loss (*p* < 0.001) and incision length (*p* < 0.001) between the two groups were statistically significant (Table 2).

**Table 2:** Surgical outcome data

	UBE (*n* = 70)	OSE (*n* = 63)	*p* Value
Hospitalization time, nights (range)	5(4–8)	6(4–8)	0.147
Operative time, min (SD)	57.6(11.9)	64.8(11.9)	0.001*
Blood loss, ml (SD)	54.0(10.4)	45.8(11.5)	< 0.001*
Fluoroscopy times (range)	3(2–5)	3(2–4)	0.110
Incision length, mm (SD)	2.7(0.3)	1.9(0.4)	< 0.001*

Data are median (range), mean (standard deviation) or number (%) as stated

*p < 0.05, statistical significance

The difference in BRA between the two groups was statistically significant (*p* < 0.05). There was no significance in the postoperative facet resection rate between the two groups (*p* > 0.05). Compared with the preoperative segments, the ROM and ST did not change obviously, with no statistical significance in each group (*p* > 0.05), and the differences between the two groups were not statistically significant (*p* > 0.05). The postoperative DH was reduced by 8.9% compared to the preoperative in each group (*p* < 0.05), and there was no significance between the two groups (*p* > 0.05). Macnab criteria were used to evaluate the clinical efficacy at 18 months after surgery, and the excellent–good rates of the UBE group and the OSE group were 88.6% and 90.5%, respectively, with no significance (*p* > 0.05) (Table 3).

**Table 3:** The comparison of the imaging relevant data of two groups

	UBE(N = 70)		OSE(N = 63)	
	Preoperative	18 months postoperatively	Preoperative	18 months postoperatively
Bone resection area(mm^2^) (SD)		109.71(25.36)		99.41(20.56)^2^
ROM (°) (SD)	5.9(1.4)	6.1(1.3)^3^	5.6(1.3)^1^	5.8(1.1)^1,3^
ST (mm) (SD)	1.3(0.8)	1.4(0.7)3	1.3(0.7)^1^	1.3(0.8)^1,3^
DH (mm) (SD)	9.0(1.0)	8.2(1.1)^4^	9.0(1.1)^1^	8.2(1.1)^1,4^
Facet resection rate, % (SD)		11.8(6.3)		11.9(6.5)^1^
Number of excellent–good cases (%)		62(88.6)		57(90.5)^1^

Mean (standard deviation) or number (%) as stated

^1^*p* > 0.05 compared with the UBE group

^2^*p* < 0.05 compared with the UBE group

^3^*p* > 0.05 compared with preoperative value in the same group

^4^*p* < 0.05 compared with preoperative value in the same group

**Fig. 2:**
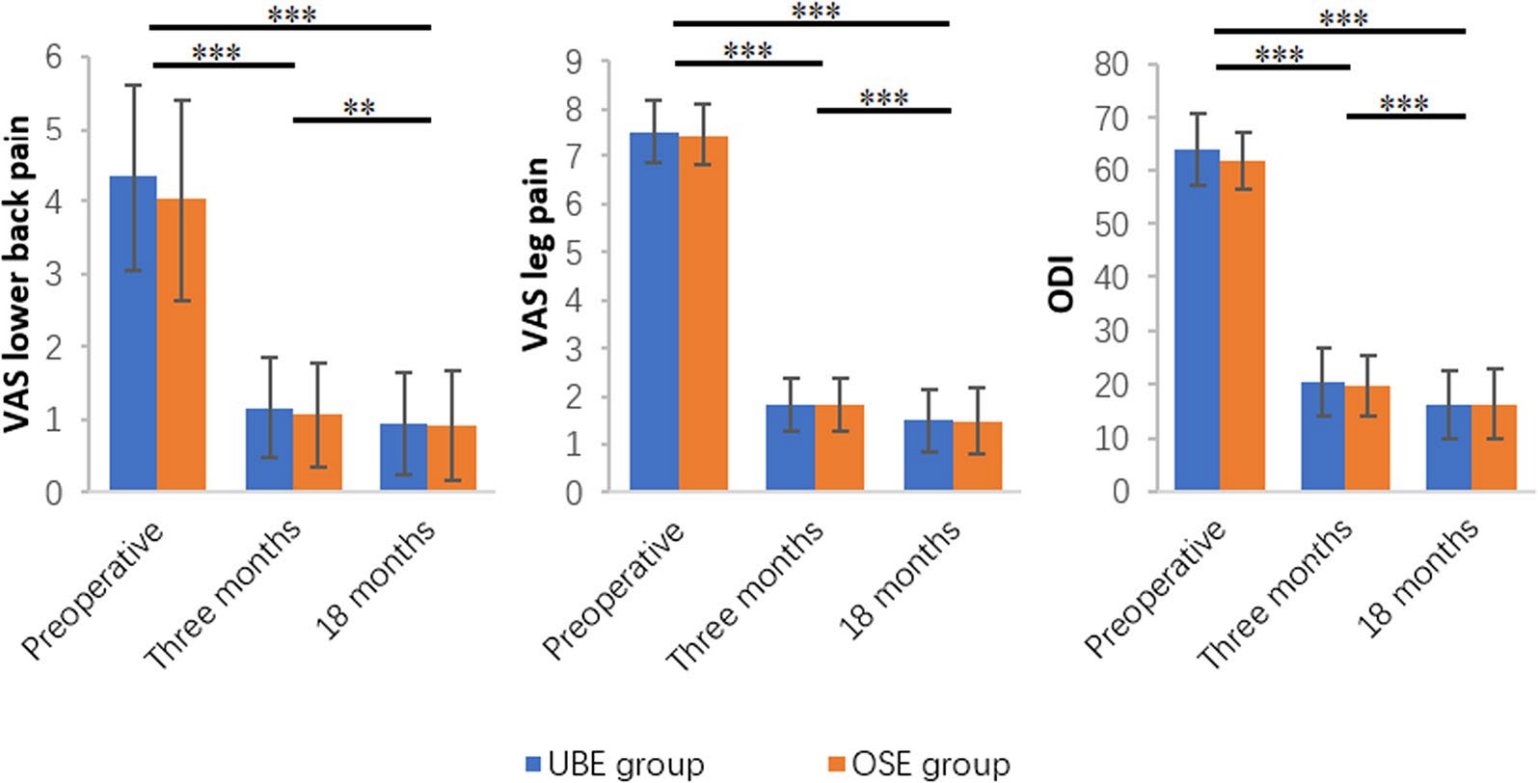
Bar charts show the results of preoperative, postoperative three months and 18 months VAS of lower back and leg pain and ODI, with the vertical line representing the SD. In either of the two groups, the comparison between any two periods was statistically significant in terms of VAS lower back pain, VAS leg pain and ODI (**p < 0.01, and ***p < 0.001)

The follow-up results of the two groups showed that the low back and leg VAS and ODI in each postoperative period were significantly lower than those before surgery, and the differences were statistically significant (*p* < 0.05). The postoperative indicators improved significantly over time, and the pairwise comparison differences were statistically significant in each group (*p* < 0.001), with no statistically significant difference between the two groups (*p* > 0.05) (Fig. 2).

### Complications

**Fig. 3:**
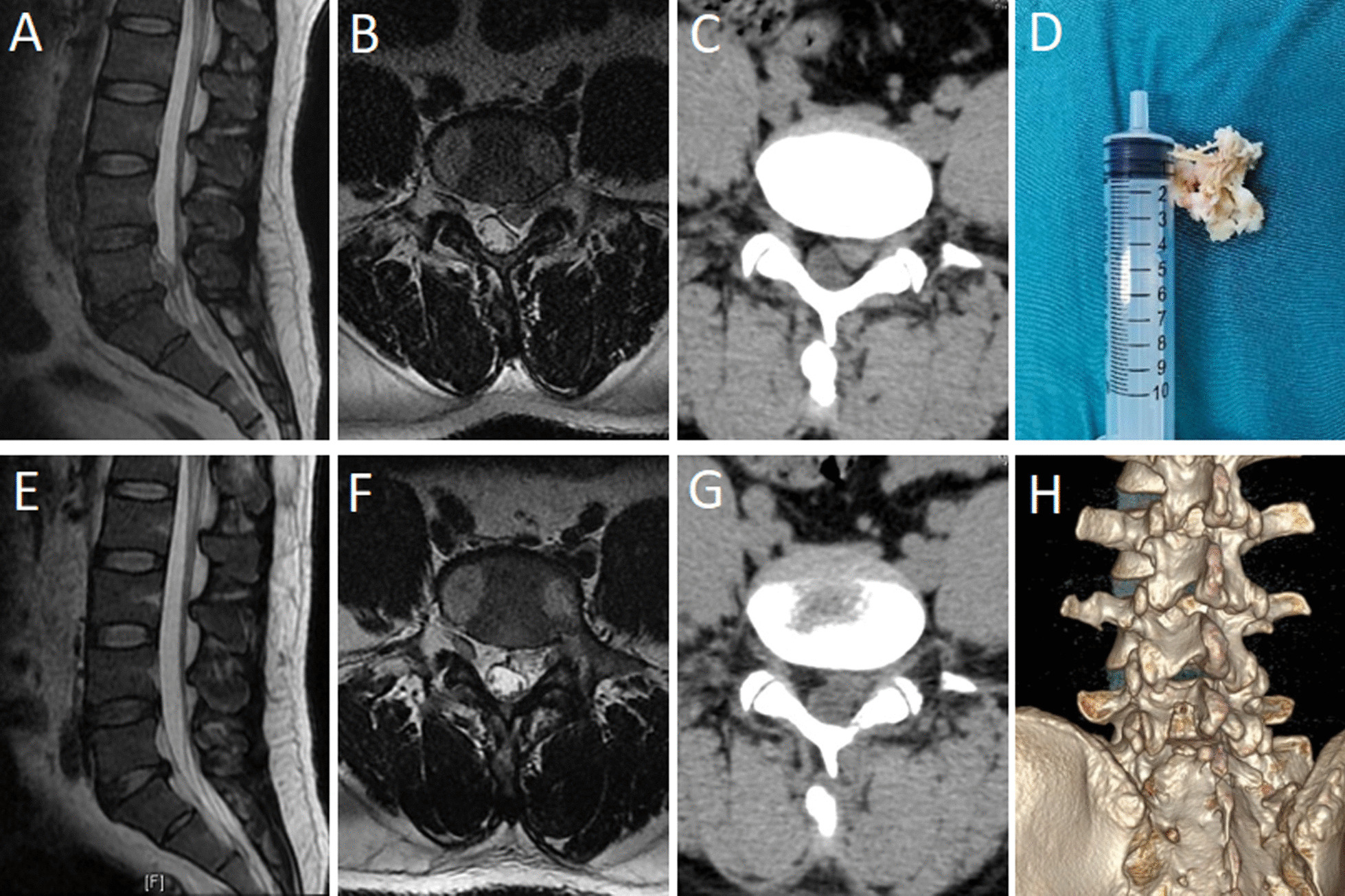
Patient, male, 53 years old, L5-S1 lumbar disk herniation, treated with UBE technology. **A**–**C** Preoperative MRI and CT showed L5-S1 intervertebral disk herniation, upward migration of the nucleus pulposus, and compression of the dura and nerve roots. **D** Removed nucleus pulposus tissue. **E**, **F** Postoperative MRI showed that migrated nucleus pulposus tissue was removed. **G**, **H** Postoperative 3D CT showed preserved facet on the surgical side and limited bone resection

**Fig. 4:**
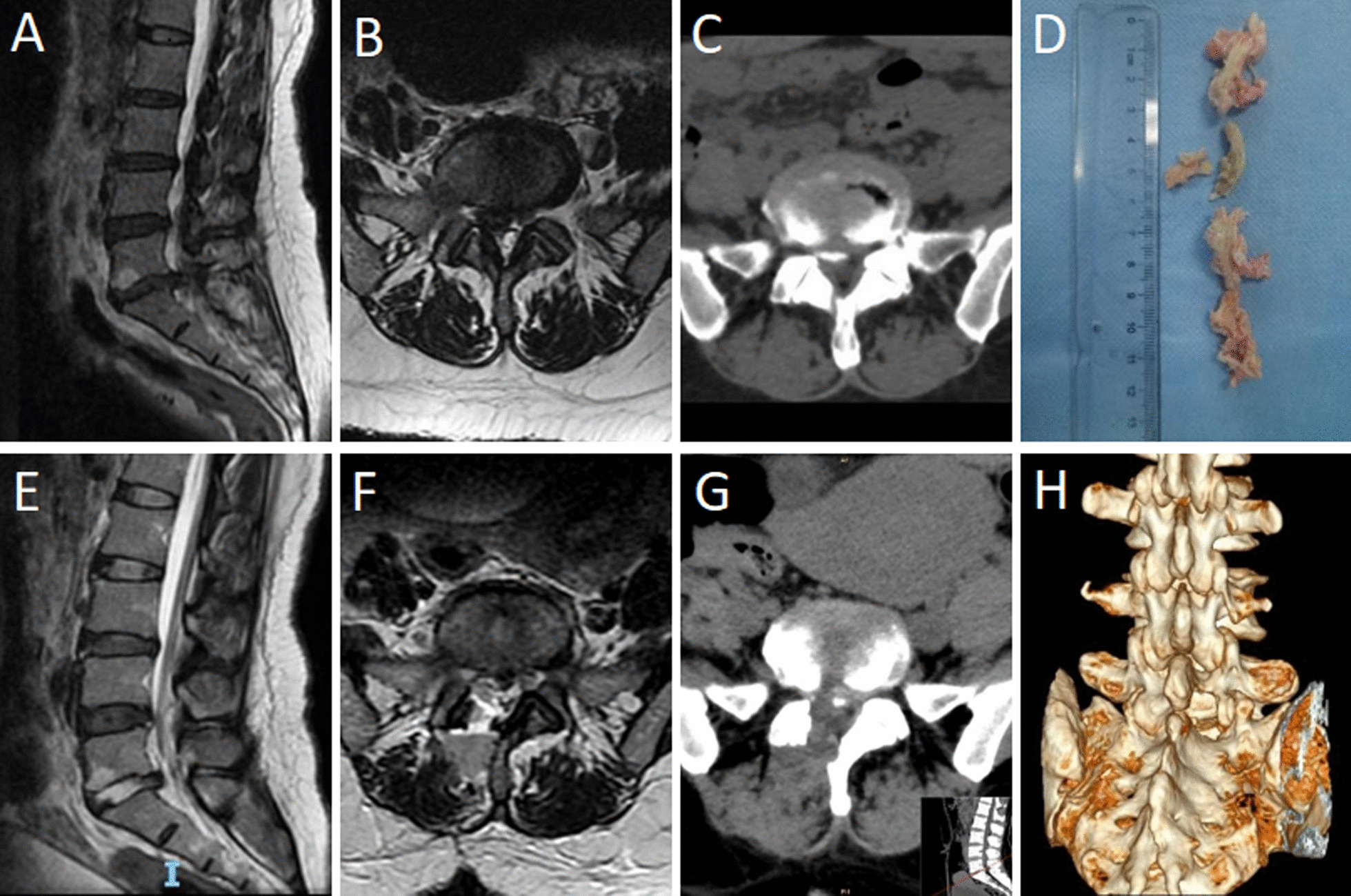
Patient, female, 50 years old, L5-S1 lumbar disk herniation, treated with OSE technology. **A**–**C** Preoperative MRI and CT showed L5-S1 intervertebral disk herniation and compression of the right S1 nerve root. **D** Removed nucleus pulposus tissue. **E**, **F** Postoperative MRI showed that nucleus pulposus tissue was removed, and the spinal canal area was significantly increased. **G**, **H** Postoperative 3D CT showed that the facet on the surgical side was preserved, the calcified tissue was partially resected, and the bone was limited resection

There was no conversion to open operation in any of the cases. In the UBE group, there was one patient with dural tear who did not receive special treatment during the operation because the tear was small. The patient’s condition was stable after one week of bed rest after surgery, and there was no cerebrospinal fluid leakage. One case of transient hypoesthesia occurred in each of the two groups after surgery, and the symptoms disappeared after nutritional neurotherapy. The postoperative incision of the two groups healed in a single stage, and there was no infection occurred. Postoperative lumbar MRI examination showed that there was no epidural hematoma formation and no residual nucleus pulposus in the spinal canal (Figs. 3 and 4). All patients were followed up for at least 18 months, with no relapse during the follow-up period.

## Discussion

The incidence of LDH is greater than 90% in L4-5 and L5-S1 intervertebral disks, because they are located at the junction of the spine and pelvis and more prone to degenerative changes and injuries than the other lumbar disks [6]. L5-S1 LDH tends to be severe in clinical presentation [7], and surgery is a choice when conservative treatment is ineffective. Traditional open surgeries, such as total laminectomy and lamina fenestration, have a wide field of view and result thorough decompression, but the incidence of spinal structural injury and symptomatic epidural scarring has been reported in the literature to exceed 10% [8]. PETD is a more classic minimally invasive treatment for LDH. However, the L5-S1 segment has peculiar anatomical features in which the transverse process space is generally narrow, the L5 vertebral arch isthmus is more lateral than that of the upper lumbar spine, and the height of the intervertebral foramen is minimal in the lumbar range [9]; additionally, other factors, such as a high iliac crest and articular hyperplasia osteophytes, make the PETD operation challenging [10]. In recent years, some surgeons have treated L5-S1 LDH with modified PELD methods, achieving satisfactory results [3], but some scholars are concerned that bony manipulation of the intervertebral foramen may increase the risk of lumbar instability after surgery [11]. The posterior anatomy of the spinal canal of the L5-S1 segment is simple; the laminar space is large, the S1 nerve root runs almost vertically, and there is only the sacral plexus nerve in the horizontal dural sac, providing a good anatomical basis for the treatment of L5-S1 LDH with the UBE technique. As an emerging minimally invasive technique in recent years, the UBE technique has many advantages in the treatment of LDH, such as wide surgical vision, flexible operation, high efficiency and satisfactory clinical efficacy [12, 13].

The OSE technique, similar to the UBE technique, has working and observation channels that can be accessed through interlaminar operations, and many scholars in China have noted its advantages when applied to clinical operations. In this study, intraoperative blood loss, incision length, and bone resection area in the OSE group were smaller than those in the UBE group, and all the patients could move the day after surgery if there were no special circumstances, thereby reducing the incidence of postoperative bedding-related complications. Excessive radiation can cause varying degrees of damage to both patients and surgeons. Ann concluded that without radiation shielding, surgeons who perform PELD 291 times per year would be exposed to the maximum permissible radiation dose [14]. In this study, the fluoroscopy times were low in both the UBE group and the OSE group, and the amount of radiation was greatly reduced. Both groups had significant improvement in VAS and ODI, and the excellent-good rates were high, indicating that the clinical efficacy of the OSE technique is comparable to that of the UBE technique. A finite element model study showed that the spinal range of motion, facet joint load, and intervertebral disk pressure increased with 30% facial resection [15]. In this study, the destruction of the facet joint was minimized during surgery. The facet resection rates in both groups were significantly less than 30%, and there was no statistical significance in the lumbar ROM and ST before and after surgery, which demonstrated that the two surgical methods protected the stability of the lumbar spine. Height loss of the intervertebral space is one of the most common radiographic findings of lumbar disk degeneration, with the intervertebral disk space narrowing progressively at an annual rate of 3.2% in women, with an average age of 50 years [16]. In this study, the change of DH before and after surgery indicated that both techniques may have a certain impact on lumbar segment degeneration.

Most scholars believe that imaging examination showing herniated disk tissue in excess of 50% of the sagittal diameter of the spinal canal can be considered giant LDH [17]. This type of herniation is large and is often combined with an inflammatory response; moreover, it easily adheres to the ligament flavum, so that the treatment by endoscopic surgery is challenging. Moreover, this type of herniation often causes severe low back and leg pain, and some patients experience bilateral neurological symptoms and even cauda equina signs [18]. It has been reported that the surgical failure rate of central LDH with giant herniation is high (15%), and percutaneous endoscopic techniques should be carefully chosen for LDH that protrudes into more than 50% of the spinal canal area [19]. In this study, there were 21 cases of giant LDH in the UBE group and 27 cases in the OSE group, all of which were thoroughly decompressed. For giant herniations, sneak decompression is performed to remove part of the ligamentum flavum, clean up the surrounding tissue of the herniation, and leave enough space to address the herniation, which can reduce the risk of nerve root or dural injury. In this study, patients with bilateral symptoms underwent “over the top” decompression treatment, and the postoperative results were satisfactory.

Migrated LDH is a more serious type of LDH and is reported to account for approximately 35–72% of LDH cases [20]. The general clinical symptoms of migrated LDH are severe, and this type is often combined with nerve root function damage or abnormalities, therefore surgery is often required when conservative treatment is not effective [1]. Lee et al. divided the prolapse site into four zones: the dissociation of the nucleus pulposus to zones two and three was called the low-grade migration type, and zones one and four were called the high-grade migration type [21]. According to the literature statistics, the high-grade migration type accounts for approximately 30% of patients with migrated LDH [22]. The UBE technique has significant clinical efficacy and is a flexible operation in the treatment of migrated LDH [9, 12]. The failure rate of PELD in high-grade migrants was reported to be as high as 15.7% [19]. We have reported that OSE has been used for different types of migrated LDH, with satisfactory clinical efficacy [23]. In this study, there were 31 cases of LDH migration in the UBE group, including 12 cases of high-grade migration type, and 22 cases in the OSE group, including 6 cases of high-grade migration type. The free nucleus pulposus tissue of all cases was completely removed during the operation, and the postoperative clinical effect was excellent, with no recurrence during the follow-up period.

The incidence of calcific lumbar disk herniation (CLDH) is approximately 4.7–15.9% [24]. PETD has some deficiencies in the treatment of L5-S1 CLDH, in which the adhesion between calcification and nerve roots or the dura mater not only increases the difficulty of PETD surgery but also may lead to iatrogenic injury [25]. Yu reported that 25 patients with CLDH were treated with PETD; the patients’ symptoms were relieved, but seven patients had postoperative dysesthesia, and one patient experienced relapse [26]. In this study, in cases of CLDH, the UBE technique and OSE technique could fully reveal and remove the calcification that compressed the nerve root from behind, isolate the nerve root, and release the stenosis caused by peripheral degeneration, with clear surgical vision, extensive exploration range, complete decompression and little postoperative nerve root injury.

Postoperative burning radicular pain or dysesthesia is a common complication after lumbar spine surgery; it is mostly transient and generally believed that it may be related to nerve root adhesion, excessive traction or compression during the surgery, or obvious hyperemia and edema of nerve roots with excessive use of bipolar radiofrequency knife. In this study, the incidence of postoperative transient hypoesthesia in the UBE group and the OSE group was 1.43% and 1.58% respectively, and the symptoms disappeared after nutritional neurological treatment, considering the possible adhesion to the nerve root. Incomplete decompression is mostly caused by deviation in preoperative judgment or deviation in the range of intraoperative decompression; it is characterized by persistent low back and leg pain and other related symptoms after surgery, which is a common reason for poor postoperative effects and affects patient satisfaction [19]. Choi et al. retrospectively analyzed 10,228 patients with LDH who underwent intervertebral foraminal surgery and found that 283 patients had decompression insufficiency [27]. There were no cases of decompression insufficiency or relapse at postoperative follow-up in this study. We think that it is important to plan reasonably, select the appropriate surgical method, and perform bilateral lateral crypt decompression if necessary. In addition, perioperative education and postoperative rehabilitation guidance are also very important to avoid recurrence. Dural tear or nerve injury is a common complication in endoscopic surgery [28]. In this study, there were no cases of dural injury in the OSE group, and there was a mild tear of the dura mater in one patient with giant LDH in the UBE group, which was considered that the large protrusion squeezed the dura chronically and made the membranous vertebral ligament adhere to the ligament flavum. Compared with the UBE technique, the microscopic field of view in the OSE technique did not have a "V" shaped angle, and the reduction of visual error may reduce the risk of dural injury.

## Conclusion

The OSE technique is an effective minimally invasive surgical option as well as the UBE technique for the treatment of L5-S1 LDH with fast localization, minimal trauma, fast recovery and satisfactory early clinical efficacy.

## Data Availability

The datasets generated during and/or analyzed during the current study are available from the corresponding author on reasonable request.

## Abbreviations

LDH: Lumbar disk herniation
OSE: One-hole split endoscopy
UBE: Unilateral biportal endoscopy
BRA: Bone resection area
ISA: Interlaminar space area
ROM: Articular process resection rate, range of motion
ST: Sagittal translation
DH: Disk height
VAS: Visual analogue score
ODI: Oswestry disability index
PELD: Percutaneous endoscopic lumbar discectomy
PETD: Percutaneous endoscopic transforaminal discectomy
BMI: Body mass index
CLDH: Calcific lumbar disk herniation
